# Supplementary material for: Exploring the Genomic Diversity and Antimicrobial Susceptibility of Bifidobacterium pseudocatenulatum in a Vietnamese Population
Source: Microbiol Spectr. 2021 Sep 15;9(2):e00526-21. doi: 10.1128/Spectrum.00526-21 (PMC8557894; doi:10.1128/Spectrum.00526-21)
Supplement: SUPPLEMENTAL FILE 2 — Supplemental material. Download SPECTRUM00526-21_Supp_1_seq10.pdf, PDF file, 0.6 MB [file spectrum00526-21_supp_1_seq10.pdf]

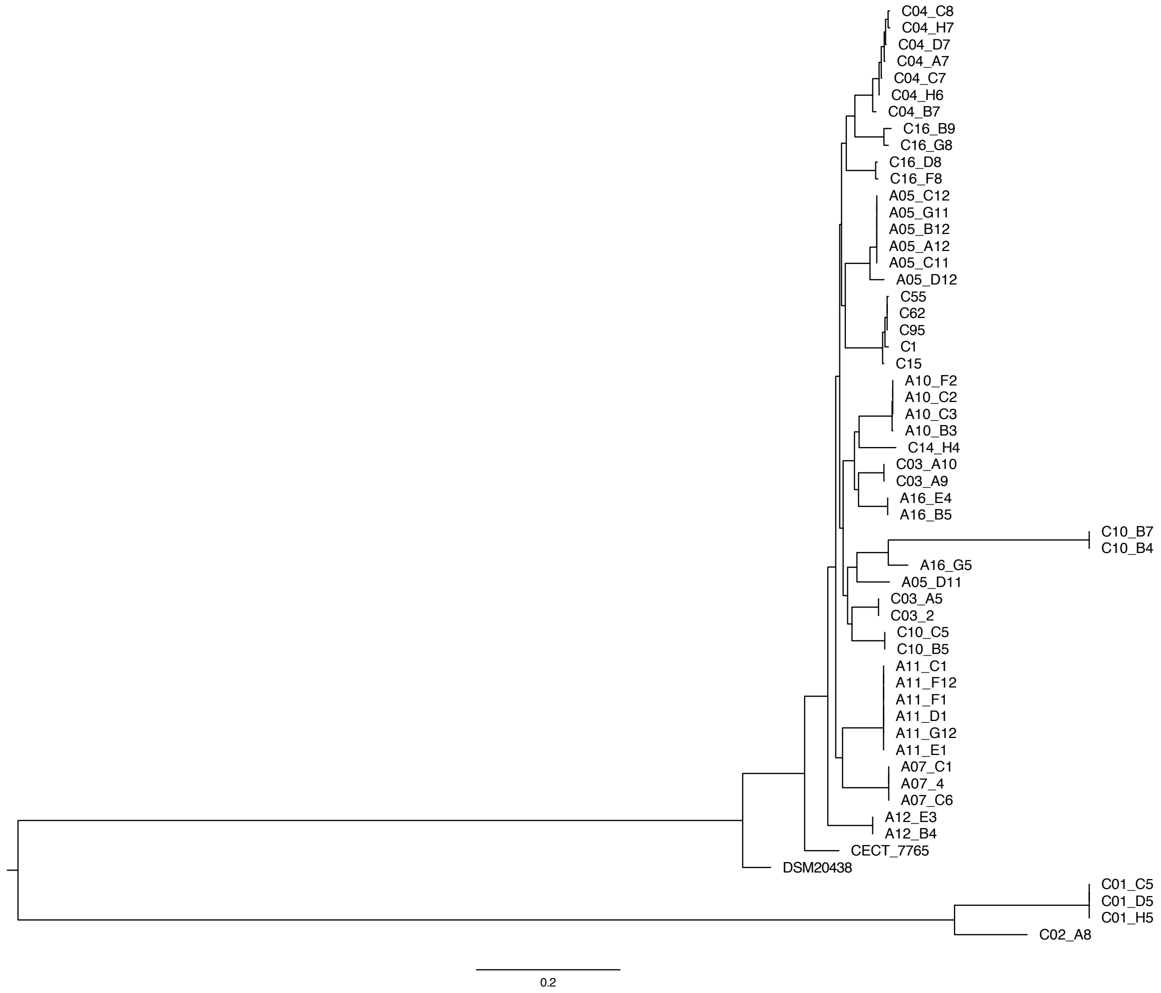

Figure S1 Phylogenetic clustering of 49 *Bifidobacterium* strains sequenced in this study and seven reference isolates. The references are *B. pseudocatenulatum* DSM20438, CECT7765 and five reconstructed assemblies from a Chinese study [1] (C1, C15, C55, C62, and C95). The maximum likelihood phylogeny was constructed based on single nucleotide polymorphism (SNP) variation in 1,116 core genes (see Methods). The horizontal scale bar denotes the number of substitutions per site.

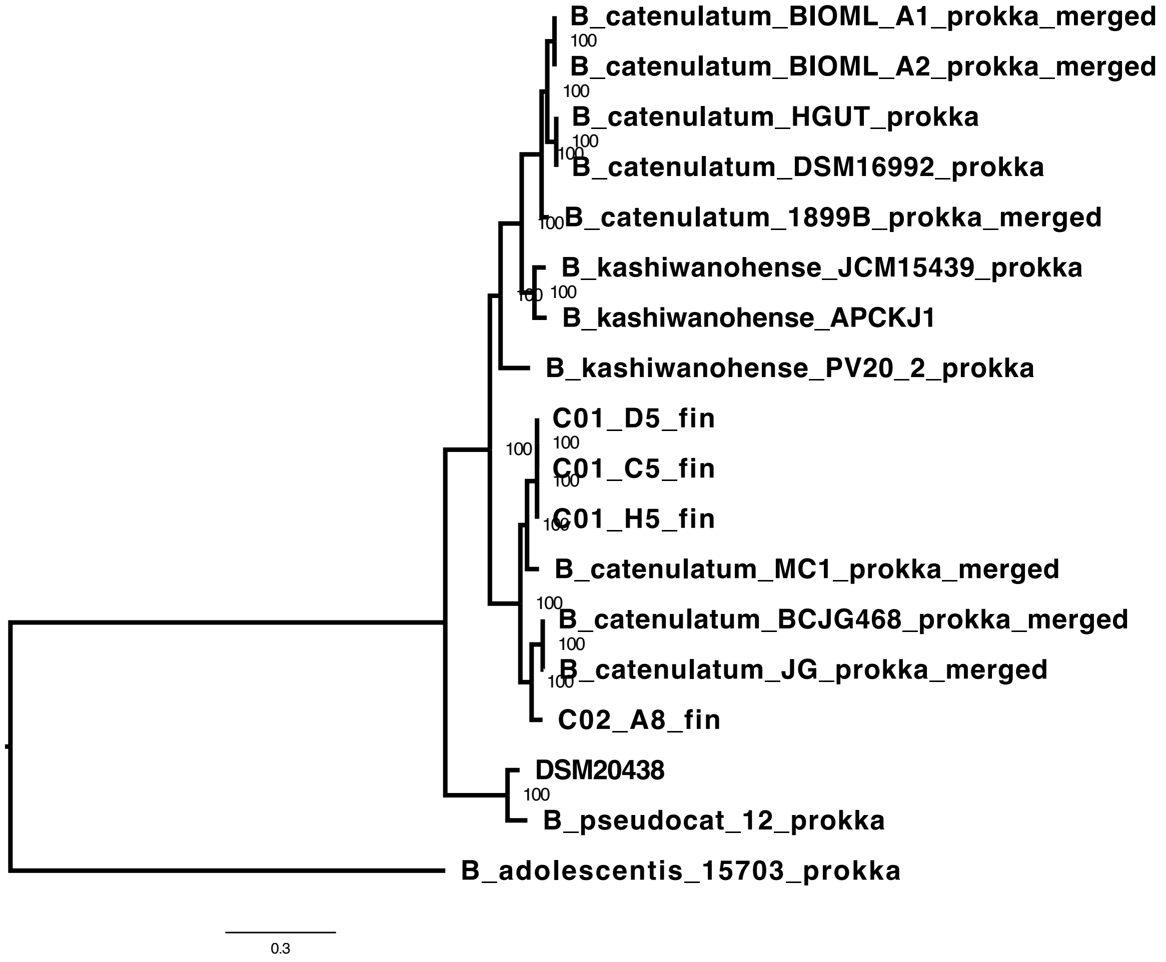

Figure S2 Phylogenetic clustering of C01 and C02 cluster *Bifidobacterium* (isolated in this study) and 14 reference sequences. The references are *B. catenulatum* (BIOML_A1, BIOML_A2, HGUT, DSM16992, 1899B, MC1, BCJG468, JG), *B. kashiwanohense* (JCM15439, APCKJ1, PV20-2), *B. pseudocatenulatum* (DSM20438, 12) and *B. adolescentis* 15703. The maximum likelihood phylogeny was constructed based on single nucleotide polymorphism (SNP) variation in 989 core genes (see Methods). The horizontal scale bar denotes the number of substitutions per site.

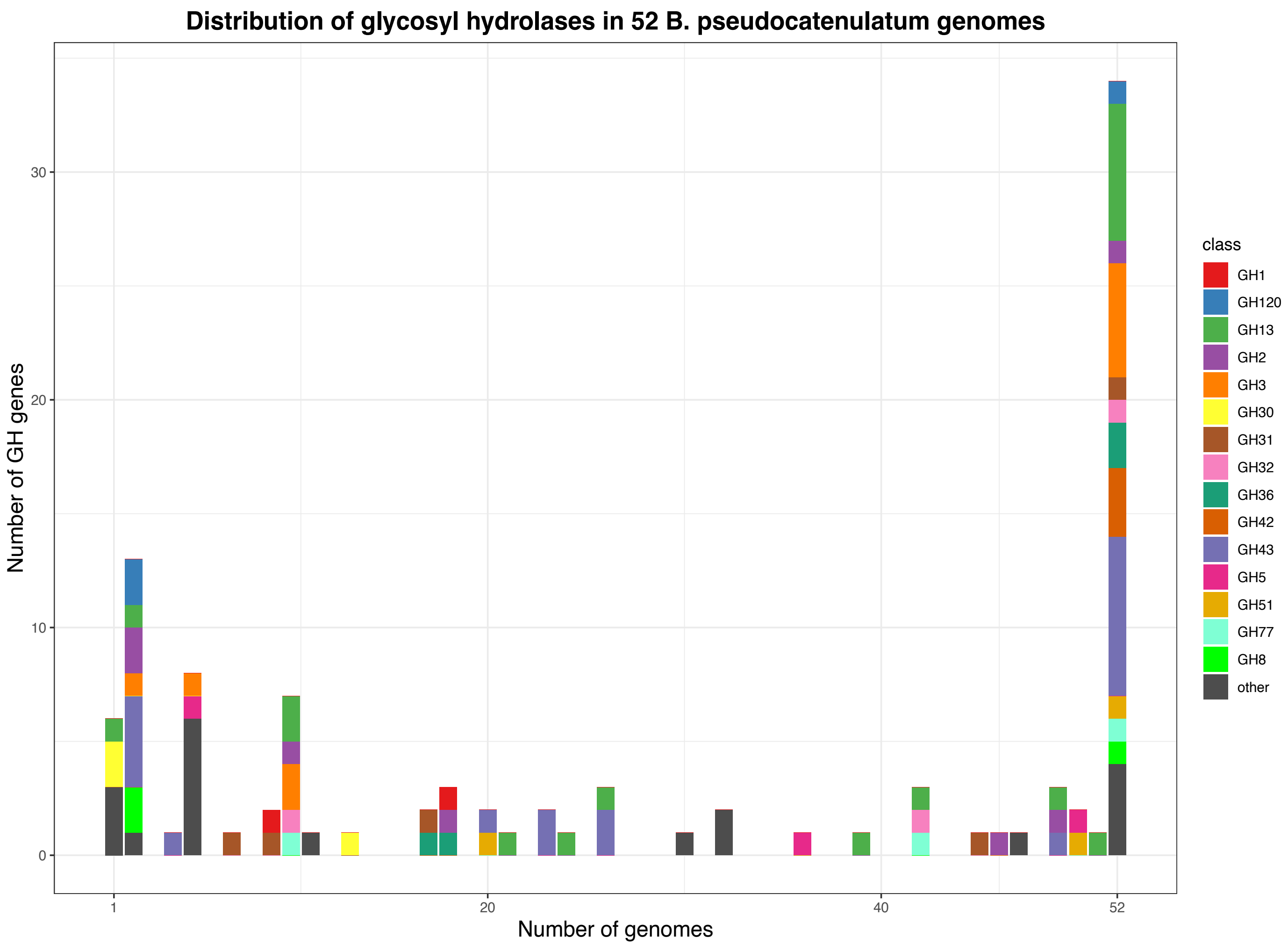

Figure S3 Distribution of glycosyl hydrolases (GH) in 52 *Bifidobacterium pseudocatenulatum* genomes (45 isolated in this study and seven reference sequences). Each bar shows the number of GH genes present in a certain number of genomes (1 – 52). Each GH gene is colored according to the GH family.

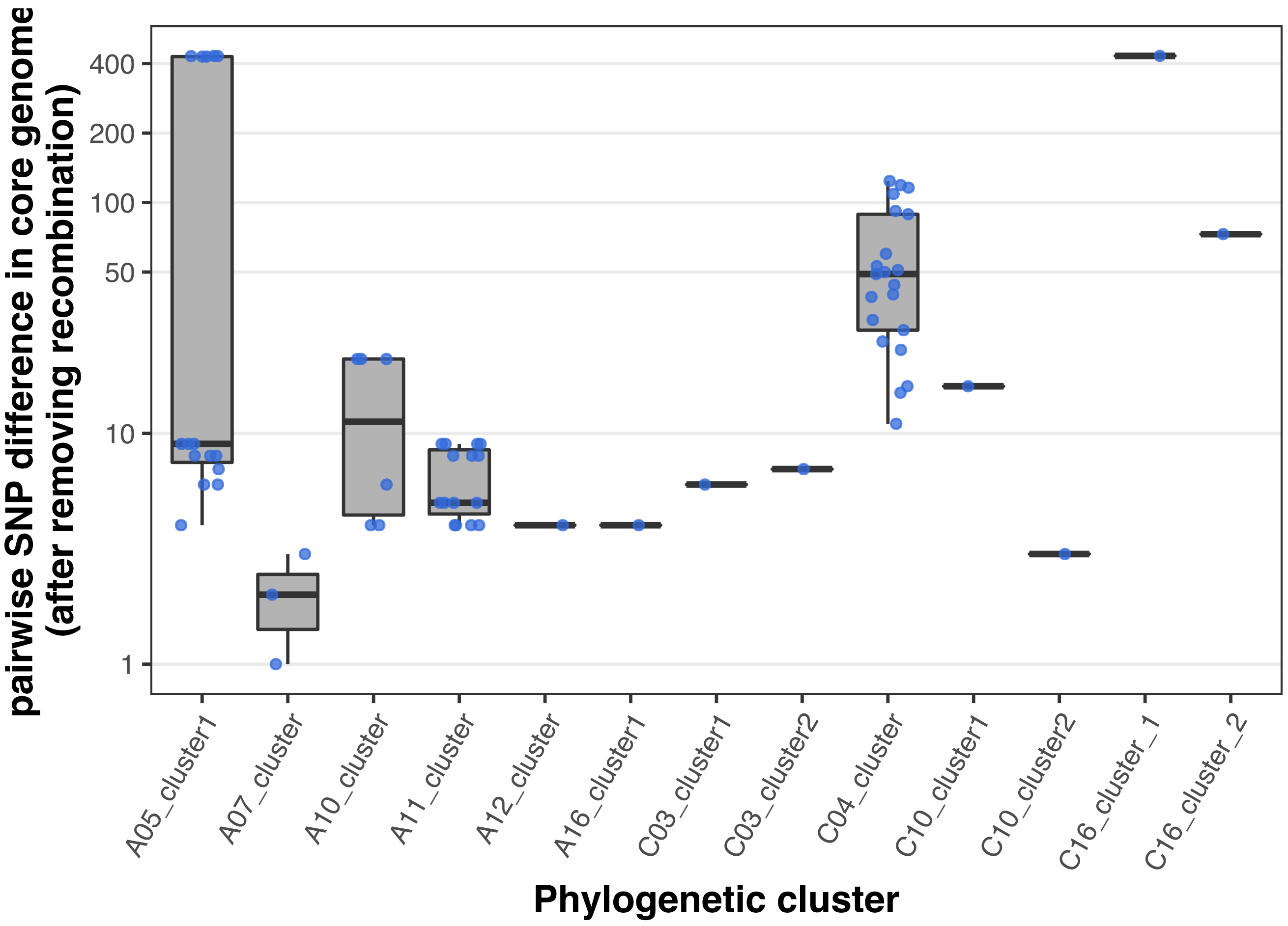

Figure S4 Variation in the core genome of Vietnamese *Bifidobacterium pseudocatenulatum*. The panel depicts the distribution of pairwise differences in recombination-free single nucleotide polymorphisms (SNPs; based on the input alignment post ClonalFrameML) of isolates within each defined phylogenetic cluster. For each boxplot, the upper whisker extends from the 75th percentile to the highest value within the 1.5 * interquartile range (IQR) of the hinge, and the lower whisker extends from the 25th percentile to the lowest value within the 1.5 * IQR of the hinge. Notice that the y-axis is in logarithmic (base 10) scale.

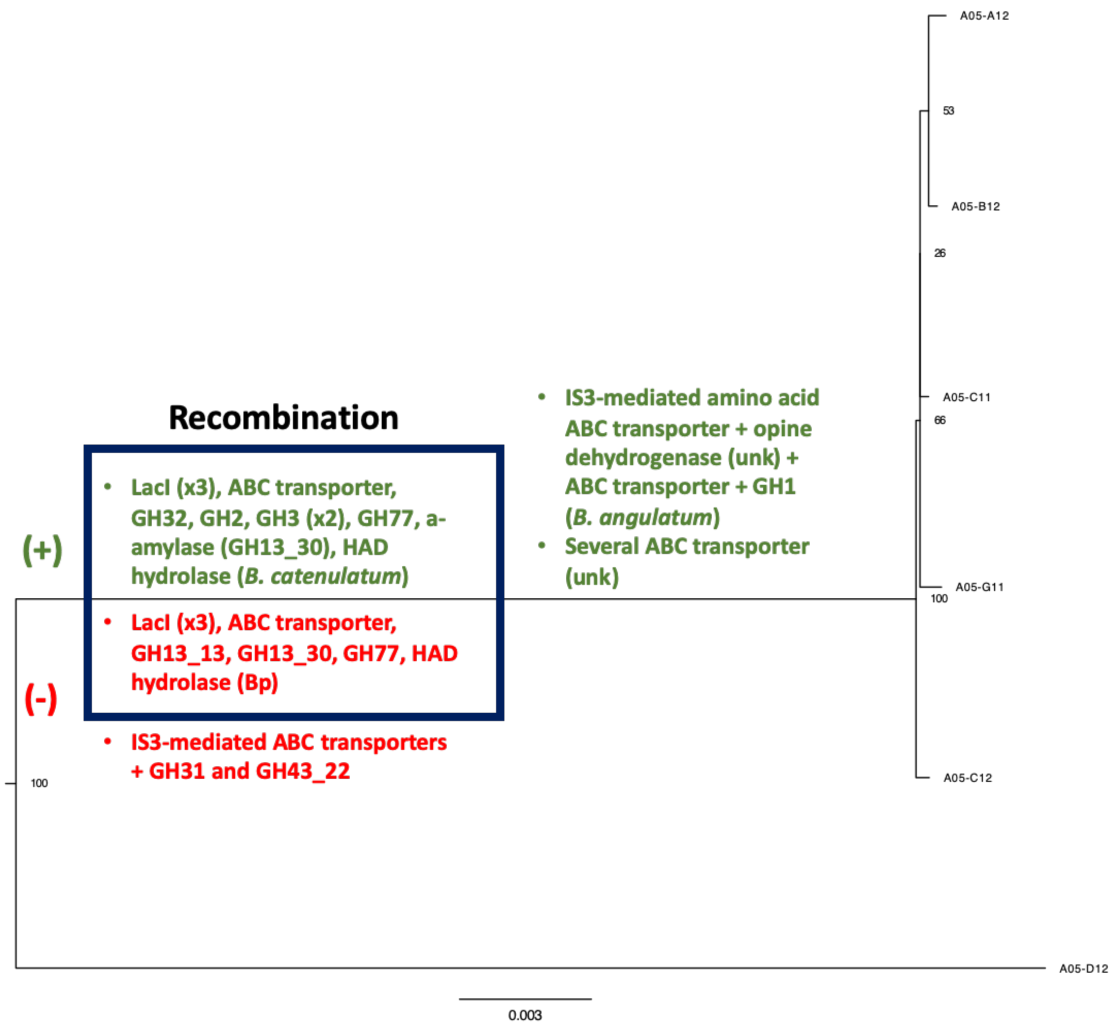

Figure S5 Clonal evolution of *Bifidobacterium pseudocatenulatum* A05_cluster. The phylogeny was a subtree derived from Figure 2. Bootstrap values are shown at the internal nodes. The tree is rooted using A05-D12 as an outgroup, as indicated by Figure 2. Genetic elements coloured in red and green represent predicted to be lost and acquired on the internal branch, respectively. The horizontal scale bar denotes the number of substitutions per site. Components inside the dark-blue box are related to a recombination event.

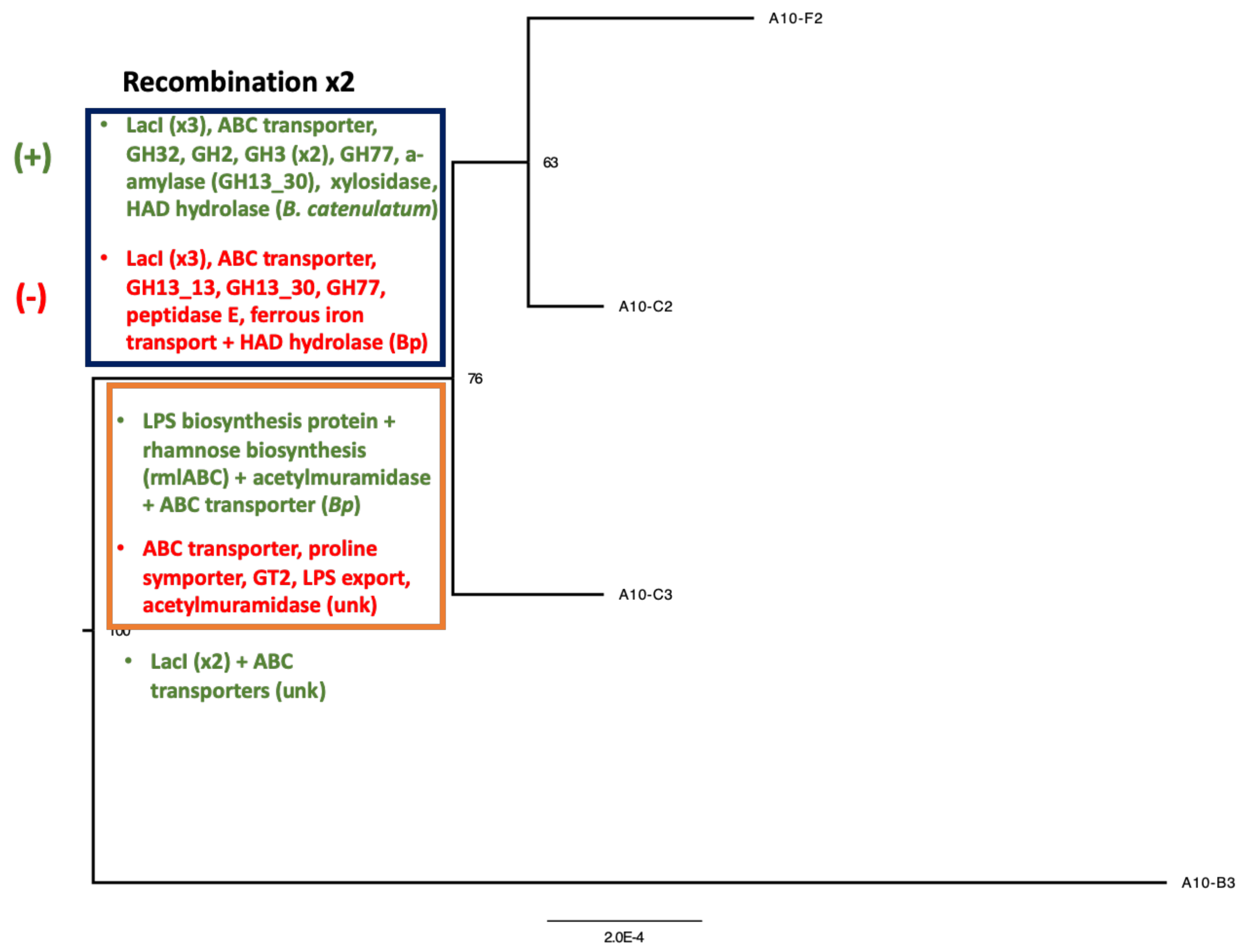

Figure S6 Clonal evolution of *Bifidobacterium pseudocatenulatum* A10_cluster. The phylogeny was a subtree derived from Figure 2. Bootstrap values are shown at the internal nodes. The tree is rooted using A10-B3 as an outgroup, as indicated by Figure 2. Genetic elements coloured in red and green represent predicted to be lost and acquired on the internal branch, respectively. The horizontal scale bar denotes the number of substitutions per site. Components inside the dark-blue and orange boxes are related to two separate recombination events.

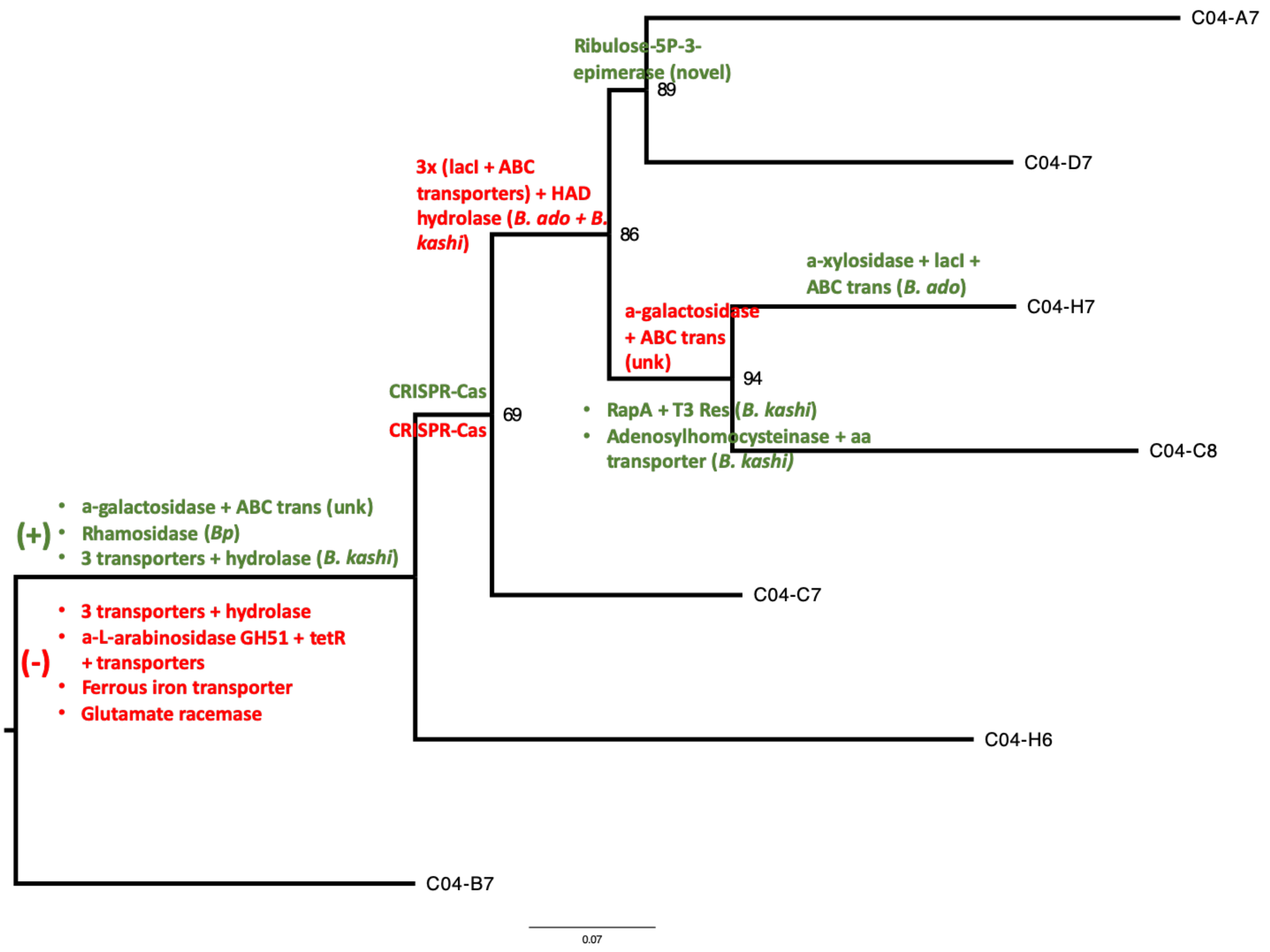

Figure S7 Clonal evolution of *Bifidobacterium pseudocatenulatum* C04_cluster. The maximum likelihood phylogeny was constructed using the recombination-free SNP alignment output by mapping (to reference DSM20438; see Methods). Bootstrap values are shown at the internal nodes. The tree is rooted using C04_B7 as an outgroup, as indicated by Figure 2. Genetic elements coloured in red and green represent predicted to be lost and acquired on the internal branch, respectively. The horizontal scale bar denotes the number of substitutions per site.

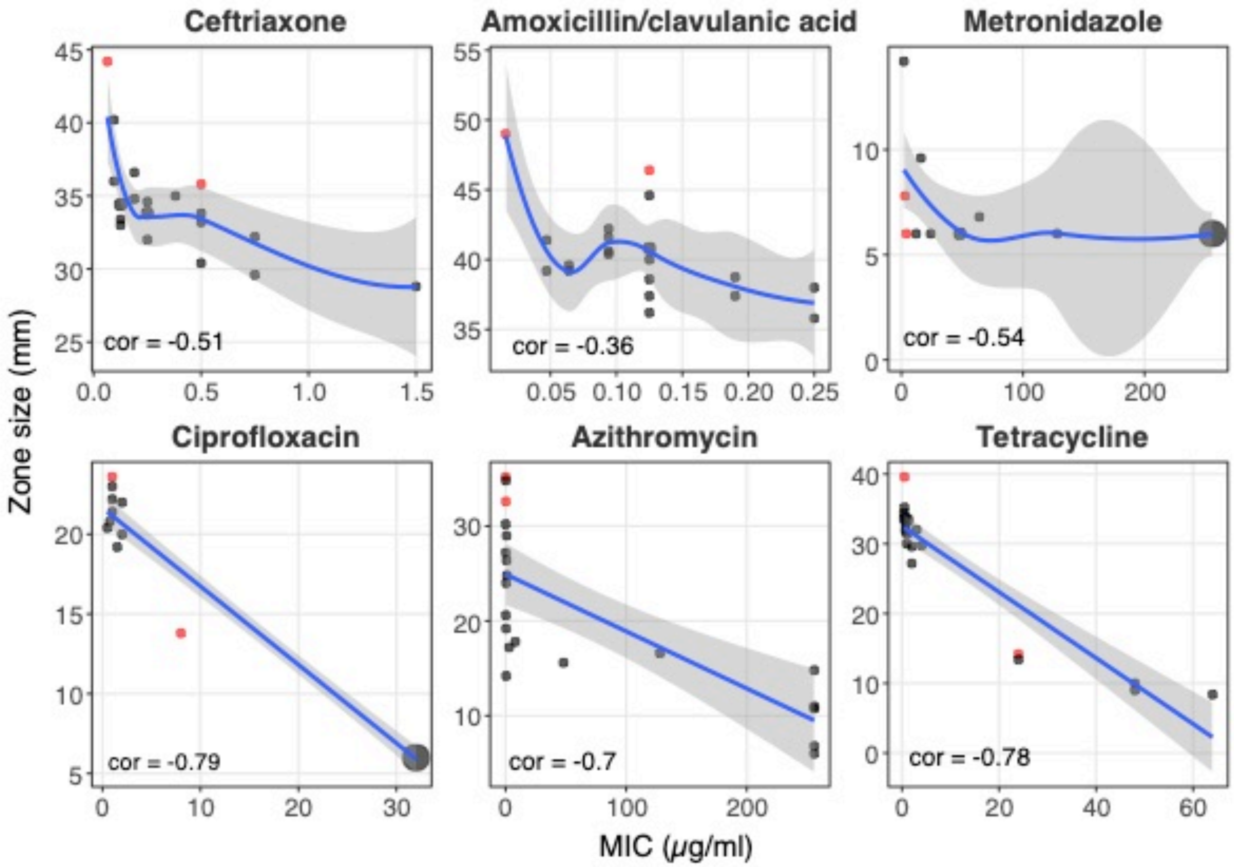

Figure S8 Correlation between E-test and disc diffusion methods in antimicrobial susceptibility testing of *Bifidobacterium*. Each panel represents a tested antimicrobial, with the x-axis and y-axis denoting the results of E-test (minimum inhibitory concentration in µg/ml) and disc diffusion (inhibitory zone diameter in mm) approaches. Controls (*B. pseudocatenulatum* DSM20438 and *B. longum* NCIMB 8809) are colored in red, while tested *Bifidobacterium* isolated in this study are colored in grey. The circle size is proportional to the number of isolates bearing the same MIC and IZD, and the largest circles in metronidazole and ciprofloxacin panels correspond to eleven isolates. All correlation scores are calculated using Kendall's correlation. LOESS regression is shown for ceftriaxone, amoxicillin/clavulanic acid, and metronidazole (cor > -0.7), while linear regression is shown for ciprofloxacin, azithromycin, and tetracycline (cor ≤ -0.7).

References

1. **Wu G, Zhang C, Wu H, Wang R, Shen J, *et al.*** Genomic Microdiversity of Bifidobacterium pseudocatenulatum Underlying Differential Strain-Level Responses to Dietary Carbohydrate. *MBio* 2017;8:1–14.
